# Simultaneous detection of l-aspartic acid and glycine using wet-chemically prepared Fe_3_O_4_@ZnO nanoparticles: real sample analysis†

**DOI:** 10.1039/d0ra03263h

**Published:** 2020-05-20

**Authors:** Mohammad Musarraf Hussain, Abdullah M. Asiri, Mohammed M. Rahman

**Affiliations:** Chemistry Department, Faculty of Science, King Abdulaziz University P. O. Box 80203 Jeddah 21589 Saudi Arabia mmrahman@kau.edu.sa mmrahmanh@gmail.com; Center of Excellence for Advanced Material Research (CEAMR), King Abdulaziz University P. O. Box 80203 Jeddah 21589 Saudi Arabia; Department of Pharmacy, Faculty of Life and Earth Sciences, Jagannath University Dhaka-1100 Bangladesh mmhussain@pharm.jnu.ac.bd m.musarraf.hussain@gmail.com

## Abstract

An easy and reliable wet-chemical method was used to synthesize iron oxide doped zinc oxide nanoparticles (Fe_3_O_4_@ZnO NPs) at a low-temperature under alkaline medium. The electrochemical characteristics of Fe_3_O_4_@ZnO NPs were investigated by using different electrochemical techniques such as UV-vis, FTIR, XRD, FESEM, XEDS, and XPS. A sensor was fabricated by deposition of a thin covering of Fe_3_O_4_@ZnO NPs onto a flat dried glassy carbon electrode (GCE) with a polymer matrix with conducting characteristics (Nafion, Nf). l-Aspartic acid and glycine were detected simultaneously by using the modified GCE/Fe_3_O_4_@ZnO NPs/Nf sensor in enzyme free conditions. Calibration curves were found to be linear for l-aspartic acid (*R*^2^ = 0.9593) and glycine (*R*^2^ = 0.8617) over a broad range of detected bio-molecule concentration (100.0 pM to 100.0 mM). The analytical sensing parameters, for example sensitivity, linear dynamic range (LDR), limit of detection (LOD), and limit of quantification (LOQ), of the proposed sensor (GCE/Fe_3_O_4_@ZnO NPs/Nf) were calculated at two potentials (+0.4 V and +0.7 V) from the calibration plot for l-aspartic acid (126.58 pM μM^−1^ cm^2^, 100.0 pM to 10.0 μM, ≈97.5 pM, and 325.0 mM) and glycine (316.46 pM μM^−1^ cm^2^, 1.0 μM to 1.0 mM, ≈13.5 pM, and 450.0 mM), respectively, by using a reliable current–voltage (*I*–*V*) technique. The synthesis of Fe_3_O_4_@ZnO NPs by means of a wet-chemical route is a good advancement for the development of doped nanomaterial based sensors from the perspective of enzyme-free detection of biological molecules in health-care areas. This proposed GCE/Fe_3_O_4_@ZnO NPs/Nf sensor was used for the particular detection of l-aspartic acid and glycine in real samples (human and rabbit serum and urine) and found to achieve reasonable and accepted results.

## Abstract 2

An easy and reliable wet-chemical method was used to synthesize iron oxide doped zinc oxide nanoparticles (Fe_3_O_4_@ZnO NPs) at a low-temperature under alkaline medium.

## Graphical abstract

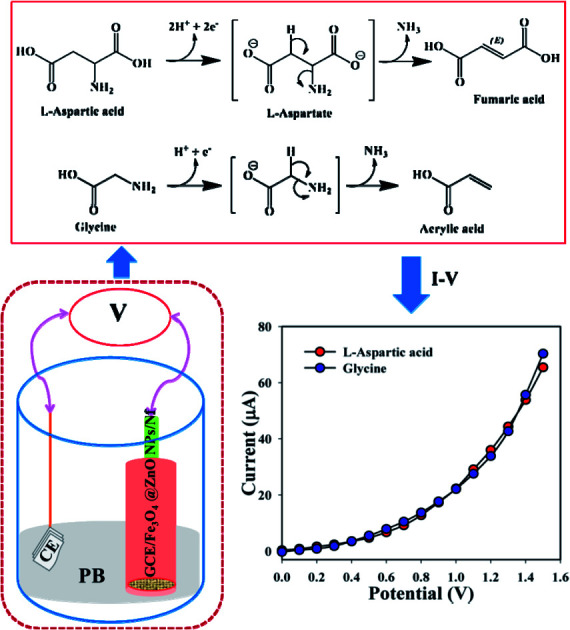

## 1. Introduction

Amino acids are significant organic molecules considered to be the biological building blocks of proteins.^1^l-Aspartic acid residues are proteins which can be isomerized by post translational modification to form d-aspartic acid (d-Asp), l-aspartic acid (l-Asp), βl-Asp, and βd-Asp.^2^ L-Asp is a non-essential amino acid which acts as a neurotransmitter. A deficiency of l-Asp can cause depression and persistent fatigue syndrome and is associated with metabolic anarchy as evidenced by neck, head, and lung cancer. An excess of l-Asp is responsible for amyotrophic lateral sclerosis, stroke, and epilepsy. Clinically, l-Asp is used as a medicine for heart disease, hepatopathy, hypertension, and as a result, a convenient sensor is needed for l-Asp detection with a wide coverage of LOD. Different techniques have been reported for the detection of l-Asp for example amperometry, capillary electrophoresis equipped flow injection, chemical oscillating systems, chiral ligand exchange capillary electrophoresis, fluorometry, ion exchange chromatography, HPLC, LCMS, potentiometry, spectrometry, and thin layer chromatography. However, these procedures are costly, solvent-intensive, and time consuming.^3–5^

Doped nanostructured materials have attracted significant interest owing to their unique properties and potential applications in chemical sensor fabrication.^6–8^ Semiconductor materials have been recognized as promising host nanomaterials for transition metals at room temperature. They have revealed a stable morphological structure and are composed of a number of irregular phases with geometrically-coordinated metals and oxide atoms, piled alternately along the axes.^9,10^ Transition metals co-doped in semiconductor nano-materials have attracted significant research interest due to their exceptional and attractive properties as well as their versatile applications.^11,12^ Recently, the demand for doped nanosized particles for electrochemical devices has increased significantly for various sensor and biosensor applications in enzyme-less detection in electrochemical approaches. Therefore, the demand for transition metal oxide doped semiconductor binary materials has increased for this purpose for the development of these sensor matrixes. Generally, metal oxide doped nanostructured materials have employed a great deal of consideration due to their chemical, structural, physical, and optical properties in terms of large-active surface areas, high-stability, high porosity, and permeability,^13,14^ which is directly dependent on their structural as well as morphological properties. Here, binary doped nanomaterials were synthesized by a facile wet-chemical method using reactant precursors in ambient conditions at low temperatures. This technique has several advantages including facile preparation and accurate control of the reactant temperature and being a one-step reaction, easy to handle, and a one-pot synthesis.^15,16^ The optical, morphological, electrical, and chemical properties of binary cladded nanostructured materials are of huge significance from the scientific aspect as well as because of the potential applications compared to other undoped nanomaterials.^17–19^ Non-stoichiometry, mostly due to oxygen vacancies, makes them conducting in nature in the doped nanostructured materials. The formation energy of oxygen vacancies and metal interstitials in semiconductors is very low and thus these defects form easily, resulting in the experimentally elevated conductivity of doped Fe_3_O_4_@ZnO nanomaterials compared to un-doped nanomaterials. Binary doped nanoparticle materials have also attracted considerable interest due to their potential applications in opto-electronics, electro-analytical techniques, selective detection of assays, sensor devices, hybrid-composites, electron-field emission sources for emission displays, biochemical detection, and surface-enhanced Raman spectroscopy *etc.*^20–22^ Therefore, doped Fe_3_O_4_@ZnO NPs offer improved sensor performances due to their large-active surface area, which increased the conductivity and current responses of the GCE/Fe_3_O_4_@ZnO NPs/NF sensor during electrochemical investigation.

Glycine (Gly) is a normal and proteinogenic amino acid broadly used as an API (active pharmaceutical ingredient) by pharmaceutical companies. Gly exists in three polymorphic forms: metastable (α), unstable (β), and thermodynamically stable (γ).^23,24^ Gly is an inhibitory neurotransmitter and a potential biomarker in brain tumors. The approximate concentration of Gly in normal adults and developing brains is 1.0 mM. An increase of Gly concentration above the WHO grade results in risk of glioblastomas and medulloblastomas.^25,26^ Based on synthesized nanomaterials a few biological molecules have been reported in previous research by Hussain *et al.*, and other researchers such as CdO·CNT NC (l-glutathione)^27^, SrO NR (l-leucine)^28^, Co_3_O_4_ nano-sheets (l-glutamic acid and uric acid)^29^, CdO nanoparticles (creatine)^30^, CuO–CdO composites (bilirubin)^31^, and ZnO·V_2_O_5_ NRs (d-glucose).^32^ Development of a novel, easy, inexpensive, and environmentally friendly sample preliminary-treatment process is in high demand at the present time for the identification, detection, confirmation, and quantification of biomolecules. This is the first report of simultaneous detection of biological molecules such as l-aspartic acid and glycine in an enzyme free environment based on iron oxide doped zinc oxide nanoparticles (Fe_3_O_4_@ZnO NPs) with a huge active surface area for adsorption of biomolecules. A new method has been proposed in this research work regarding enzyme free detection of l-aspartic acid and glycine by using a GCE/Fe_3_O_4_@ZnO NPs/Nf modified sensor as a mediator in the *I*–*V* method at ambient conditions.

## 2. Experimental section

### 2.1 Materials and methods

Reagent grade chemicals for example EtOH, Nafion (Nf), NaOH, acetylcholine, ascorbic acid, l-aspartic acid, cholesterol, choline, l-cysteine, dopamine, GABA, glycine, lactic acid, testosterone, l-tyrosine, and uric acid were obtained from Sigma-Aldrich, KSA and used as received. UV-visible spectra and FTIR of the ZnO NPs, Fe_3_O_4_ NPs, and Fe_3_O_4_@ZnO NPs were recorded on a UV-visible spectrophotometer (Thermo scientific) and an FTIR spectrometer (Thermo scientific, NICOLET iS50, Madison, USA), correspondingly. Electrochemical criteria such as arrangement, morphology, particle size, and elemental investigation of the nanoparticles (ZnO NPs, Fe_3_O_4_ NPs, and Fe_3_O_4_@ZnO NPs) were also examined using FESEM (JEOL, JSM-7600F, Japan) equipped with XEDS. XPS analyses were carried out for the purpose of binding energy determination among Zn, Fe, and O on a K-α1 spectrometer. Powder XRD experiments were also carried out to determine the crystalline pattern of the NPs. The *I*–*V* method was conducted at an exacting point using the modified GCE/Fe_3_O_4_@ZnO NPs/Nf sensor by means of an electrometer (Keithley, USA) in order to detect biological molecules.

### 2.2 Synthesis of Fe_3_O_4_@ZnO NPs

**Scheme 1:**
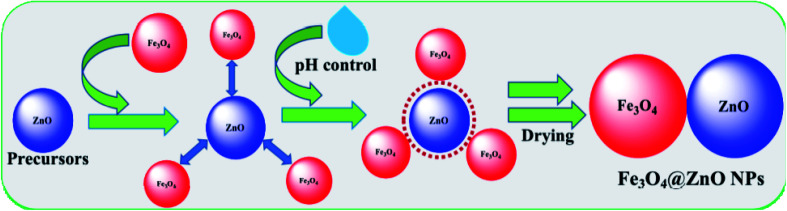
Fabrication mechanism of Fe_3_O_4_@ZnO NPs.

Zinc chloride (ZnCl_2_), ferrous sulfate (FeSO_4_·7H_2_O), and NaOH were used as the reacting agents in the preparation of Fe_3_O_4_@ZnO NPs using a simple wet-chemical technique.^33,34^ A wet-chemical procedure is a recognized solid state system and is extensively used in the production of un-doped or doped nano-materials and will attain yields of minor grains as well as shorter periods of phase growth. Based on this process, ZnCl_2_ (13.79 g) and FeSO_4_·7H_2_O (13.90 g) were dissolved in distilled water in two separate round bottom flasks for the preparation of a stock solution of ZnCl_2_ (100.0 mM and 1.0 L) and FeSO_4_ (100.0 mM and 500.0 mL) under continuous stirring. Doped Fe_3_O_4_@ZnO NPs (100.0 mL) were made from these stock solutions in 1 : 1, 1 : 2, 1 : 3, and 1 : 4 ratios. The resulting solutions were pH controlled by adding NaOH (Table 2) and then placed on a hot plate at 90.0 °C with continual stirring. Following 6.0 h of constant stirring, the flasks were washed thoroughly with water and acetone consecutively and afterward kept in open air (24.0 h) at room temperature for solvent evaporation. The derived Fe_3_O_4_@ZnO NPs were desiccated in the oven at 60.0 °C (25.0 h), ground into powders, and dried for a second time at 60.0 °C in the oven (24.0 h) successively in order to be used for electrochemical categorization and application. A feasible approach for the development of Fe_3_O_4_@ZnO NPs is presented in reactions ((i)–(v)) and Scheme 1.

i ZnCl_2_ + 2NaOH → Zn(OH)_2(aq)_ + 2NaCl_(s)_↓

ii Zn(OH)_2_ → ZnO_(s)_↓ + H_2_O

iii FeSO_4_ + 2NaOH → Fe(OH)_2(aq)_ +Na_2_SO_4(s)_↓

iv 3Fe(OH)_2_ → Fe_3_O_4(s)_↓ + H_2_O + H_2_↑

v Fe_3_O_4_ + ZnO → Fe_3_O_4_@ZnO_(s)_↓

### 2.3 Preparation and fabrication of GCE with nanoparticles

A set of phosphate buffer (PB) solutions (100.0 mM and pH = 5.7, 6.5, 7.0, 7.5, and 8.0) were prepared from NaH_2_PO_4_, Na_2_HPO_4_, and distilled water (Table S1†). First, the GCE was washed thoroughly with distilled water and acetone, respectively, and then kept in the open air to dry (1.0 h). The NPs (ZnO, Fe_3_O_4_, and Fe_3_O_4_@ZnO) were dispersed in EtOH to make a slurry which was then deposited on the dried surface of the GCE and kept in the open air to dry (1.0 h). A coating binder (Nafion, Nf) was added dropwise with the dried deposited NPs and kept again in the open air (2.0 h) to complete drying with a uniform film formation. The fabricated GCE and platinum (Pt) wire were used as the working and counter electrodes, respectively, with the purpose of illuminating the electrochemical responses by using the *I*–*V* practice towards the proposed sensor (GCE/Fe_3_O_4_@ZnO NPs/Nf) regarding detection of biomolecules.

## 3. Results and discussion

### 3.1 Analysis of optical properties

Optical features are one of the considerable unique properties for the assessment of the photo-catalytic movement of the NPs (ZnO, Fe_3_O_4_, and Fe_3_O_4_@ZnO). According to the UV-visible spectroscopy hypothesis, the spectra and band-gap energies (BE) of the metal oxide can be achieved due to the adsorption of radiant energy throughout the shifting of the external electrons of the atom to the higher energy state. UV-visible spectra of the ZnO NPs, Fe_3_O_4_ NPs, and Fe_3_O_4_@ZnO NPs were performed in the 200–800 nm range and broad absorption bands were achieved (Table 1). From the highest level of band absorption, the theoretical BE of the ZnO NPs, Fe_3_O_4_ NPs, and Fe_3_O_4_@ZnO NPs was calculated based on eqn (vi). According to Tauc's equation (direct band gap rule, eqn (vi)–(ix)), the practical BE of the ZnO NPs, Fe_3_O_4_ NPs, and Fe_3_O_4_@ZnO NPs was calculated. *hv vs.* (*αhv*)^2^ was plotted and subsequently extrapolated to the *x*-axis. From the extrapolated curve, the practical BE for the ZnO NPs, Fe_3_O_4_ NPs, and Fe_3_O_4_@ZnO NPs was found (Fig. S1, S2† and Table 1). Here, *α* = absorption coefficient, *h* = Planck's constant, *v* = frequency, *r* = 0.5 (direct transition), and *A* = constant related to the effective mass of the electrons.^35^vi
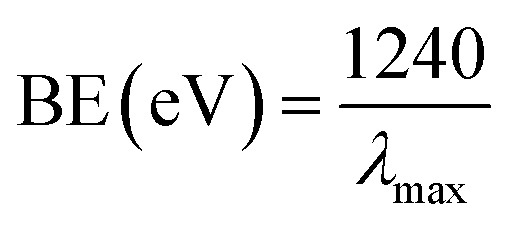

vii *hν* = *A*(*αhν*)^1/*r*^

viii *hν* = *A*(*αhν*)^2^

ix *hν* ∞ (*αhν*)^2^

**Table 1:** *λ*_max_ and band gap energies of the nanoparticles

Nanoparticles		Physical state	pH	*λ*_max_ (nm)	Band gap energy (eV)	
					Theoretical	Practical (Tauc's equation)
ZnO NPs		White	10.23	367.0	3.38	3.0
Fe_3_O_4_ NPs		Black	10.32	298.7	4.15	2.9
Fe_3_O_4_@ZnO NPs	1 : 1	Red	10.26	266.8	4.65	3.2
	1 : 2	Brown	10.35	300.2	4.13	3.3
	1 : 3	Brown	10.32	300.0	4.13	3.5
	1 : 4	Black	10.29	298.1	4.16	3.6

### 3.2 Structural evaluation

FTIR spectra were recorded (4000–400 cm^−1^) with the purpose of identification of the functional nature of the ZnO NPs, Fe_3_O_4_ NPs, and Fe_3_O_4_@ZnO NPs under normal conditions regarding atomic and molecular properties. The reported peaks at 1407, 1165, 870, and 555 cm^−1^ were assigned to the existence of C–H, Zn–O–Fe, C–H, and Zn=O in the NPs, respectively (Fig. S3†). Rational peaks at 870 and 555 cm^−1^ were denoted for the understanding of the metal-oxide (Zn–O–Fe) bond which recognized the design of the Fe_3_O_4_@ZnO NPs.

A crystallite background is the implication of a metal–oxygen skeleton. In order to make a distinction of the crystallite nature of the prepared ZnO NPs, Fe_3_O_4_ NPs, and Fe_3_O_4_@ZnO NPs, XRD investigation was conducted in the range of 2*θ* = 10–80°. Potential peak intensity with an indication for 2*θ* originated at 200, 100, 111, 220, 311, 222, 422, 511, 440, and 533 degrees (Fig. S3†). All the realistic peaks in the spectra were assigned according to the JCPDS file (019-0629, 36-1451 and 41-1426).^36–38^ Average crystallite sizes and lattice strains of the nanoparticles were calculated using the Scherrer equation (*x* and Table 2)^39^, where *D*_p_ = average crystallite size (nm), *λ* = X-ray wavelength (Å), *β* = line broadening (radian), and *θ* = Bragg's angle (°).x
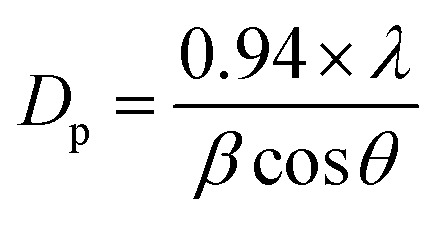


**Table 2:** Crystallite size and lattice strain of the nanoparticles^a^

NPs		*β* (°)	*β* (rad)	2*θ* (°)	*θ* (°)	cos *θ*	*λ* (Å)	CS (nm)	LS
ZnO NPs		0.04	0.698 × 10^−3^	36.34	18.17	0.950	1.54	218.31	0.5 × 10^−3^
Fe_3_O_4_ NPs		0.3	5.236 × 10^−3^	37.86	18.93	0.946		29.23	3.8 × 10^−3^
Fe_3_O_4_@ZnO NPs	1 : 1	0.11	1.920 × 10^−3^	35.16	17.58	0.953		79.11	1.5 × 10^−3^
	1 : 2	0.02	0.349 × 10^−3^	25.66	12.83	0.975		425.42	0.4 × 10^−3^
	1 : 3	0.03	0.524 × 10^−3^	32.60	16.30	0.960		287.77	0.4 × 10^−3^
	1 : 4	0.04	0.698 × 10^−3^	35.60	17.80	0.952		217.85	0.5 × 10^−3^

a NPs = nanoparticles, *β* = line broadening, 2*θ* = peak position, *θ* = Bragg's angle, *λ* = X-ray wavelength, CS = crystallite size, and LS = lattice strain.

### 3.3 Morphological and elemental analysis

**Fig. 1:**
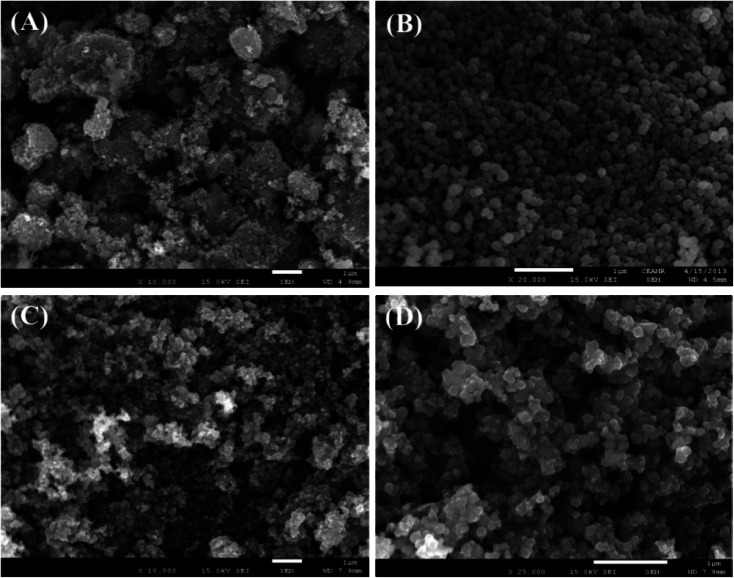
Magnified FESEM images of (A) ZnO NPs, (B) Fe_3_O_4_ NPs, and (C and D) Fe_3_O_4_@ZnO NPs.

FESEM is an effective method to study the morphological properties of nanoparticles. The morphology and elemental nature of the ZnO NPs, Fe_3_O_4_ NPs, and Fe_3_O_4_@ZnO NPs were investigated by FESEM. The characteristic morphology of the ZnO NPs, Fe_3_O_4_ NPs, and Fe_3_O_4_@ZnO NPs was recorded at a low to high exaggerated range (Fig. 1). According to the XEDS examination, zinc (Zn), oxygen (O), and iron (Fe) exist in the Fe_3_O_4_@ZnO NPs. A comparison in weight (%) among the ZnO NPs, Fe_3_O_4_ NPs, and Fe_3_O_4_@ZnO NPs is presented in Table 3. No supplementary peaks were found regarding impurities in the FESEM and XEDS spectra which recognized that the nanoparticles were composed of Zn, Fe, and O (Fig. S4†).

**Table 3:** Weight (%) and binding energies of the nanoparticles

Nanoparticles		Weight (%)			Binding energies (eV)				
		O	Zn	Fe	O 1s	Fe^2+^		Zn^2+^	
						2p_3/2_	2p_1/2_	2p_3/2_	2p_1/2_
ZnO NPs		42.07	57.93	—	532.0, 535.6	—	—	1023	1046
Fe_3_O_4_ NPs		6.93	—	93.07	537.2	718	—	—	—
Fe_3_O_4_@ZnO NPs	1 : 1	33.46	25.77	19.63	534.0	715	728	1025	1048
	1 : 2	35.45	23.87	40.68	532.2	713	725	1022	1045
	1 : 3	37.45	38.07	24.18	530.3	711	724	1021	1044
	1 : 4	33.10	49.06	17.84	529.6	710	724	1021	1044

### 3.4 Assessment of the binding energy

**Fig. 2:**
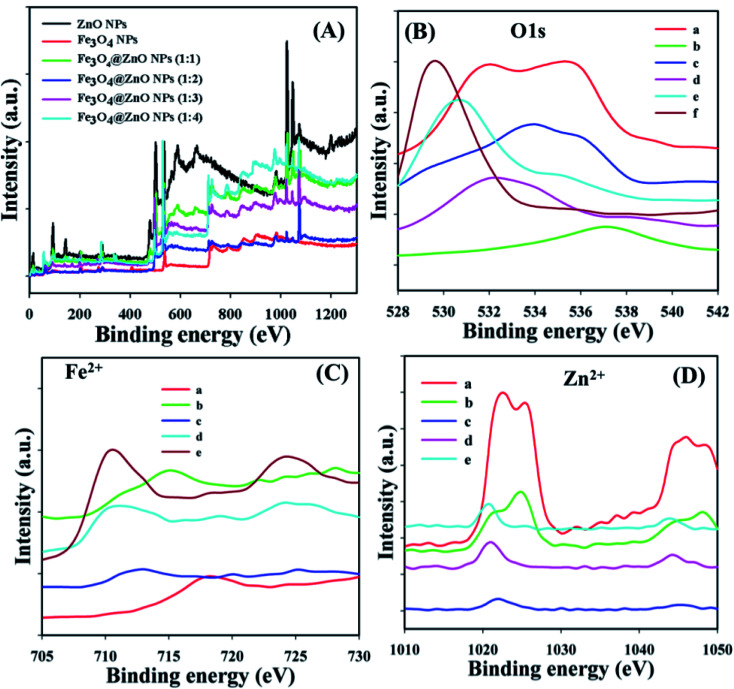
Binding energy study, (A) full spectrum, (B) a: ZnO NPs, b: Fe_3_O_4_ NPs, and c–f: Fe_3_O_4_@ZnO NPs (1 : 1–1 : 4), (C) a: Fe_3_O_4_ NPs, (D) a: ZnO NPs, (C and D) b–e: Fe_3_O_4_@ZnO NPs (1 : 1–1 : 4).

X-ray photoelectron spectroscopy (XPS) is a quantitative spectroscopic experiment utilized to determine the chemical background of the fundamentals present in the Fe_3_O_4_@ZnO NPs. Kinetic force including the electron number of a sample may be estimated by irradiation of an X-ray beam with NPs during XPS examination. Usually, the empirical formula, elemental-composition, and the chemical as well as electronic nature of fundamentals existing in a material can be evaluated using this procedure. Based on the XPS analysis, zinc, iron, and oxygen were present in the prepared Fe_3_O_4_@ZnO NPs (Fig. 2A). The O 1s, spin–orbit Fe^2+^, and Zn^2+^ spectra were assigned to the main peaks which determined that oxygen (O^2−^), iron (Fe^2+^), and zinc (Zn^2+^) were present in the NPs (Table 3 and Fig. 2B–D).^40–42^

## 4. Applications

### 4.1 Simultaneous detection of l-aspartic acid and glycine by Fe_3_O_4_@ZnO NPs

**Scheme 2:**
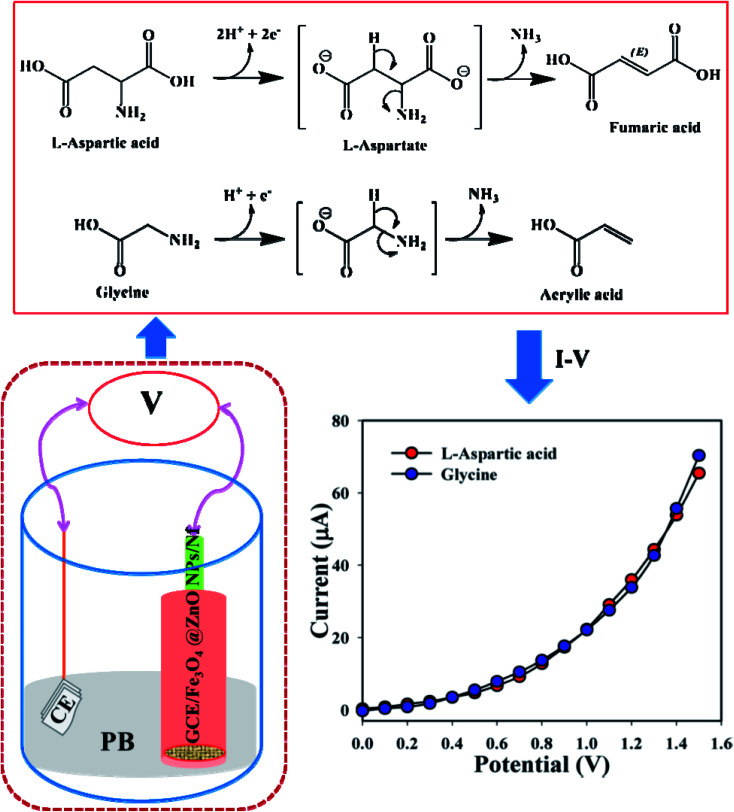
Electrode setup and possible electrochemical mechanism for the enzyme free detection of l-aspartic acid and glycine.

The development of a fabricated electrode with nanoparticles is the foundation of utilizing a sensor. Fe_3_O_4_@ZnO NPs bear numerous advantages such as being chemically stable, simple to fabricate, non-toxic, safe, and have broad exposure with a huge active surface area in air. Depending on the adsorption of biomolecules, GCE/Fe_3_O_4_@ZnO NPs/Nf was characterized as a modified sensor for the recognition and sensitive and selective determination of biological molecules such as l-aspartic acid and glycine in phosphate buffer. A possible mechanism for the detection of l-aspartic acid and glycine by using the GCE/Fe_3_O_4_@ZnO NPs/Nf sensor based on *I*–*V* practice is presented in Scheme 2. Here, l-aspartic acid was converted into an intermediate product l-aspartate, releasing two electrical particles (protons and electrons). After that, l-aspartate was also converted into fumaric acid with the release of ammonia. On the other hand, glycine was converted into acrylic acid with the release of one electrical particle (a proton and electron) and ammonia as an intermediate product. These released electrons are responsible for the current–voltage response towards the projected sensor (GCE/Fe_3_O_4_@ZnO NPs/Nf) for bio-molecule (l-aspartic acid and glycine) detection.^29^

**Fig. 3:**
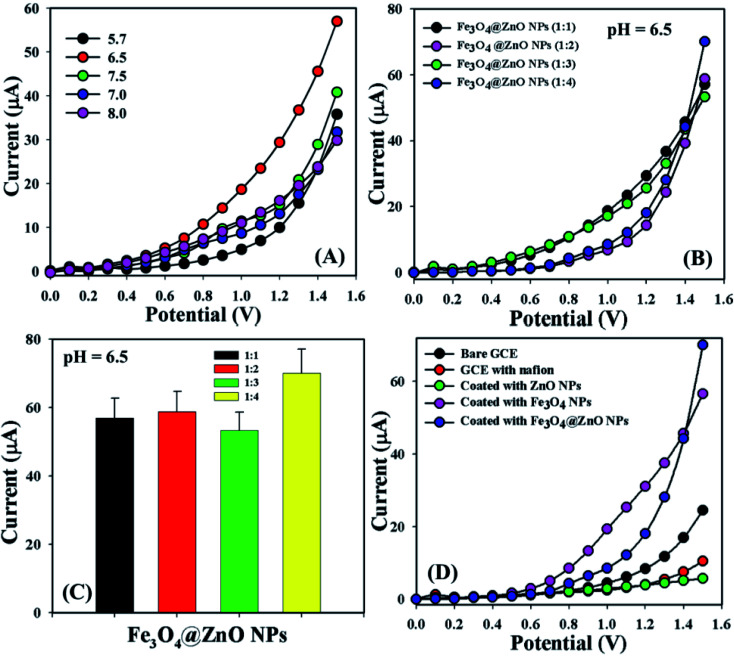
(A) pH examination, (B) nanoparticle ratios optimization at pH: 6.5, (C) bar diagram presentation of NP optimization at +1.5 V with error limit 10.0%, and (D) electrochemical responses of the different modified electrodes.

Bio-molecular agents that are significantly useful in health care fields were categorized by using a sensor (GCE/Fe_3_O_4_@ZnO NPs/Nf). Phosphate buffer solutions with different pH values from mildly acidic to basic (5.7, 6.5, 7.0, 7.5, and 8.0) were optimized to distinguish which method was more suitable to sensitively and selectively detect the biomolecules. In this regard, pH = 6.5 was found to be more rapid to respond in the *I*–*V* dimension (Fig. 3A). The NP ratio of Fe_3_O_4_@ZnO was optimized in the perspective of pH = 6.5 to identify which ratio was more appropriate and Fe_3_O_4_@ZnO NPs (1 : 4) was found to be more responsive in this regard to selectivity detect the biomolecules (Fig. 3B). Fig. 3C is the bar diagram presentation of the Fe_3_O_4_@ZnO NPs ratio optimization at +1.5 V with an error limit of 10.0%. *I*–*V* responses for the uncoated GCE and the GCE coated with Nafion, ZnO NPs, Fe_3_O_4_ NPs, and Fe_3_O_4_@ZnO NPs on the working electrode were investigated in PB with pH = 6.5. The differences of the current responses among the GCE occurred because the current signals were increased by Fe_3_O_4_@ZnO NPs compared with other modified GCEs (Fig. 3D).

**Fig. 4:**
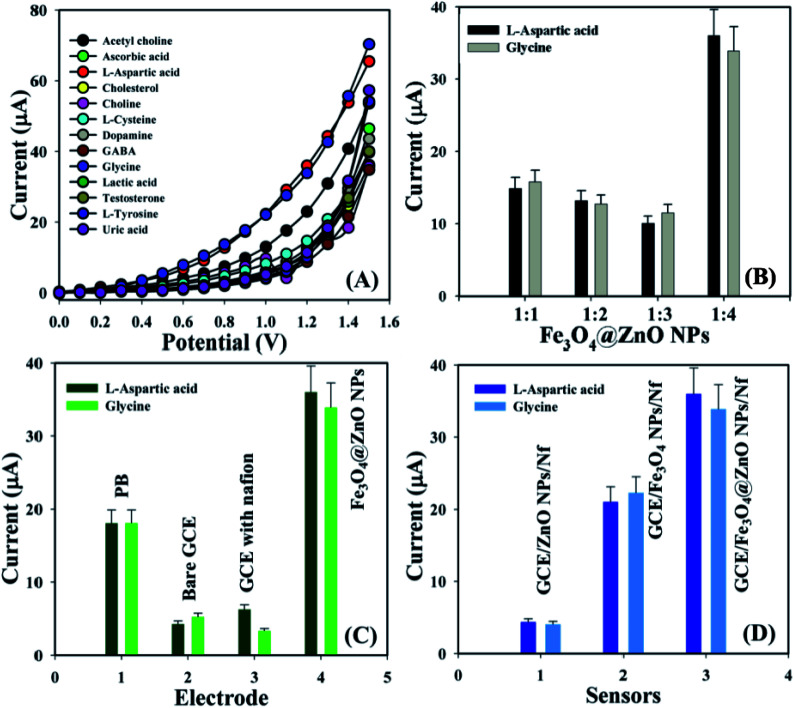
(A) Selectivity examination, (B) selectivity optimization at +1.2 V with error bar 10.0%, (C) absence and presence of biomolecules at +1.2 V with error border 10.0%, and (D) control experiment at +1.2 V with error limit 10.0%.

Biological molecules such as acetylcholine, ascorbic acid, l-aspartic acid, cholesterol, choline, l-cysteine, dopamine, GABA, glycine, lactic acid, testosterone, l-tyrosine, and uric acid were examined at a concentration of 1.0 μM in order to find out the maximum current responses towards the GCE/Fe_3_O_4_@ZnO NPs/Nf sensor and consequently it was noticeably observed that the modified sensor was more selective towards l-aspartic acid and glycine compared with other biomolecules (Fig. 4A). Selectivity was optimized at 1.0 μM concentration in perspective of the Fe_3_O_4_@ZnO NPs ratios and Fe_3_O_4_@ZnO NPs (1 : 4) appeared foremost in their responses towards l-Asp and Gly, respectively. Fig. S5† and 4B is the bar diagram presentation of selectivity optimization at +1.2 V with error limit 10.0%. An increase in the current responses was reported with regards to the GCE/Fe_3_O_4_@ZnO NPs/Nf sensor with l-Asp and Gly which has given an enormous surface area with better exposure in absorption and adsorption potentiality compared with other modified sensors (Fig. S6†). Fig. 4C is the bar diagram presentation of the absence and presence of the biomolecules at +1.2 V with error bar 10.0%. A control experiment at 1.0 μM was conducted and the GCE/Fe_3_O_4_@ZnO NPs/Nf sensor showed more responses towards l-aspartic acid and glycine compared with GCE/ZnO NPs/Nf and GCE/Fe_3_O_4_ NPs/Nf (Fig. S6†). Fig. 4D is the bar diagram illustration of the control experiment at +1.2 V with error limit 10.0%.

**Fig. 5:**
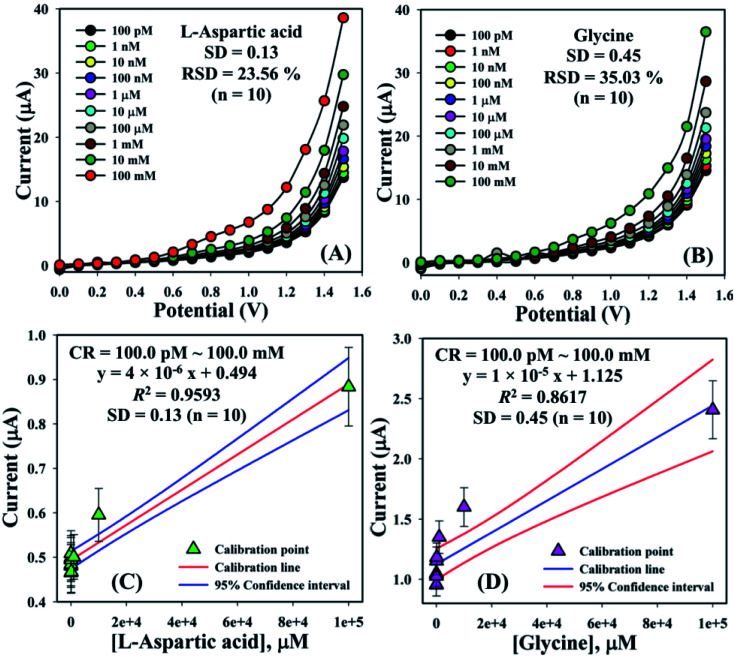
Concentration variation studies for {(A) l-aspartic acid and (B) glycine} and calibration plots {(C) l-aspartic acid (at +0.4 V) and (D) glycine (at +0.7 V) with error limit 10.0%}.

The electrical responses of the l-aspartic acid and glycine towards the GCE/Fe_3_O_4_@ZnO NPs/Nf sensor were examined with an indication of the current changes of the modified electrodes which was a function of the biological molecule concentration under normal conditions. It was observed that the current responses of the preferred biomolecules increased frequently from lower to higher concentrations of l-aspartic acid (SD = 0.13, RSD = 23.56%, and *n* = 10) and glycine (SD = 0.45, RSD = 35.03, and *n* = 10), respectively (Fig. 5A and B). A good range of the bio-molecule concentrations was examined from lower to higher potentials (0.0–+1.5 V) in order to find out the apparent analytical limit. Calibration curves at +0.4 and +0.7 V were plotted from a range of biomolecules concentrations (100.0 pM to 100.0 mM) and were found to be linear, l-aspartic acid (*R*^2^ = 0.9593) and glycine (*R*^2^ = 0.8617) (Fig. 5C and D). The sensitivity, LOD, and LOQ were calculated from the calibration plot using the eqn (xi)–(xiii) for l-aspartic acid (126.58 pM μM^−1^ cm^2^, ≈97.5 pM, and 325.0 mM) and glycine (316.46 pM μM^−1^ cm^2^, ≈13.5 pM, and 450.0 mM), respectively.^43–45^ Here, *m* = slope of the calibration curves (*y* = 4 × 10^−6^*x* + 0.494 and *y* = 1 × 10^−5^*x* + 1.125), *A* = active surface area of the GCE (0.0316 cm^2^), and SD = standard deviation (0.13 and 0.45 at *n* = 10) of l-aspartic acid and glycine towards the proposed sensor at the calibrated potentials (+0.4 and +0.7 V), respectively.xi
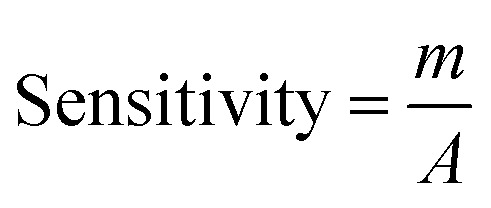
xii
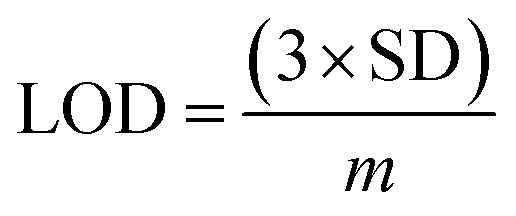
xiii
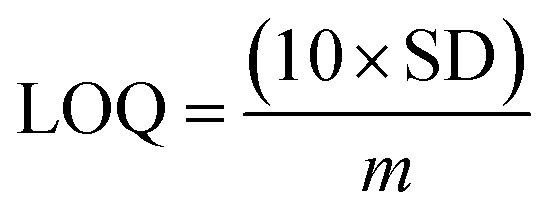


**Fig. 6:**
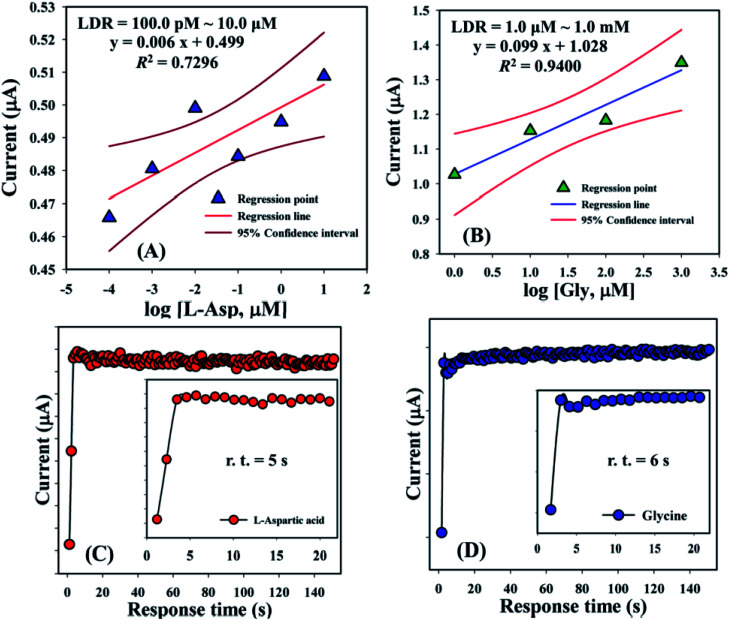
Linear dynamic range and response time curve for (A and C) l-aspartic acid and (B and D) glycine. Inset: response time (r.t.) expansion (0–20 s).

Linear dynamic range curves were also calculated from the calibrated plot and found to be linear for l-aspartic acid (100.0 pM to 10.0 μM, *y* = 0.006*x* + 0.499, and *R*^2^ = 0.7296) and glycine (1.0 μM to 1.0 mM, *y* = 0.099*x* + 1.028, and *R*^2^ = 0.9400), correspondingly (Fig. 6A and B). The response times of l-aspartic acid and glycine towards the GCE/Fe_3_O_4_@ZnO NPs/Nf modified sensor were determined at 1.0 μM and found to be 5.0 and 6.0 s, respectively (Fig. 6C and D). A comparison of l-aspartic acid and glycine detection using different modified electrodes is presented in Table 4. It was clearly noticed that one of the significant analytical sensing parameters (sensitivity) of the proposed sensor is calculated in our research work. However, this parameter was not found in the previous reported research work.

**Table 4:** Determination of l-aspartic acid and glycine using different modified electrodes^a^

BM	Modified electrodes	Methods	LDR (μM)	LOD (μM)	Sensitivity (pA μM^−1^ cm^−2^)	Ref.
l-Asp	Imprinted P13AA	DPASV	0.15–8.9	0.016	—	3
	Copper nanoparticles	FI	300.0–700.0	30.0	—	46
	Polypyrrole	ME	7.5–60.0	7.5	—	47
	Polypyrrole	ME	7.5–100	3.0	—	48
	Imprinted carboxybetaine polymer	SP	(5.0–15.0) × 10^3^	—	—	49
	GCE/Fe_3_O_4_@ZnO NPs/Nf	*I*–*V*	1.0–1.0 (μM to mM)	97.5 mM	126.58	This work
Gly	NiONPs/GCE	AM	1–200	0.9	24.3 nA μM^−1^	50
	Paste electrode	CV + SWSV	5–60 ng L^−1^	0.65 ng L^−1^	—	51
	GCE/Fe_3_O_4_@ZnO NPs/Nf	*I*–*V*	100.0–10.0 (pM to μM)	135.0 mM	316.46	This work

a BM = biomolecules, DPASV = differential pulse anodic stripping voltammetry, FI = flow injection, ME = microelectrode, SP = spectrometry, AM = amperometry, CV = cyclic voltammetry, SWSV = square-wave stripping voltammetry, l-Asp: l-aspartic acid, Gly: glycine, and *I*–*V* = current–voltage.

### 4.2 Examination of sensor skill

**Fig. 7:**
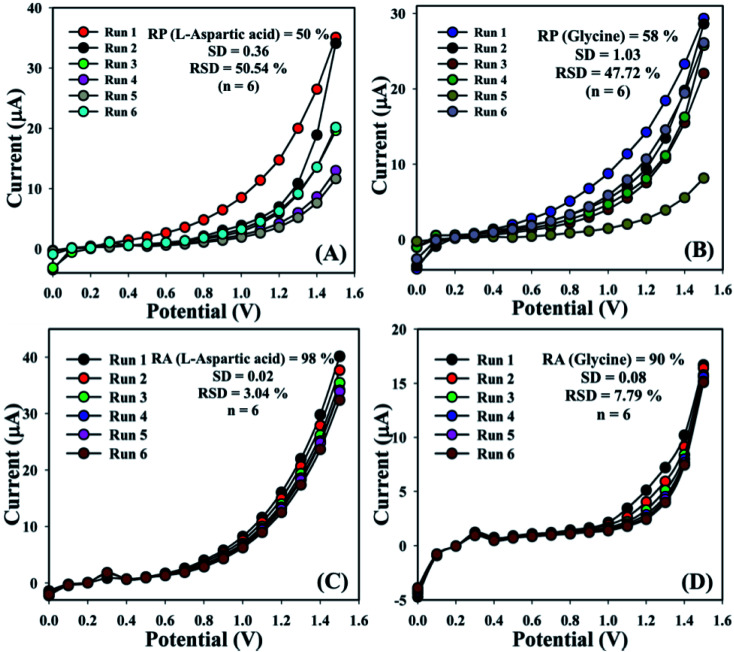
Reproducibility and repeatability study for (A and C) l-aspartic acid and (B and D) glycine.

The sensing aptitude of the GCE/Fe_3_O_4_@ZnO NPs/Nf sensor was investigated up to a few days for the evaluation of the reproducible (RP) abilities. In view of that, a series of six consecutive experiments of l-aspartic acid and glycine concentrations (1.0 μM) were performed and yielded good reproducible responses at a calibrated potentials (+0.4 and +0.7 V) with the proposed sensor in an unusual environment, (l-aspartic acid: RP = 50%, SD = 0.36, RSD = 50.54%, and *n* = 6 and glycine: RP = 58%, SD = 1.03, RSD = 47.72%, and *n* = 6) (Fig. 7A, B and Table S2†). It was acknowledged that the current responses were not largely changed after washing of each experiment of the fabricated GCE/Fe_3_O_4_@ZnO NPs/Nf sensor.

The storage capacity of the GCE/Fe_3_O_4_@ZnO NPs/Nf modified sensor was evaluated under regular conditions at 1.0 μM. In this regard, a series of six successive experiments of the sensor were performed and yielded good repeatability (RA) at calibrated potentials (+0.4 and +0.7 V) such as l-aspartic acid (RA = 98%, SD = 0.02, RSD = 3.04%, and *n* = 6) and glycine (RA = 90%, SD = 0.08, RSD = 7.79%, and *n* = 6) (Fig. 7C, D and Table S3†). It was clearly shown that this proposed sensor could be used without any significant problems with sensitivity up to a few days.

### 4.3 Study of interference effects

**Fig. 8:**
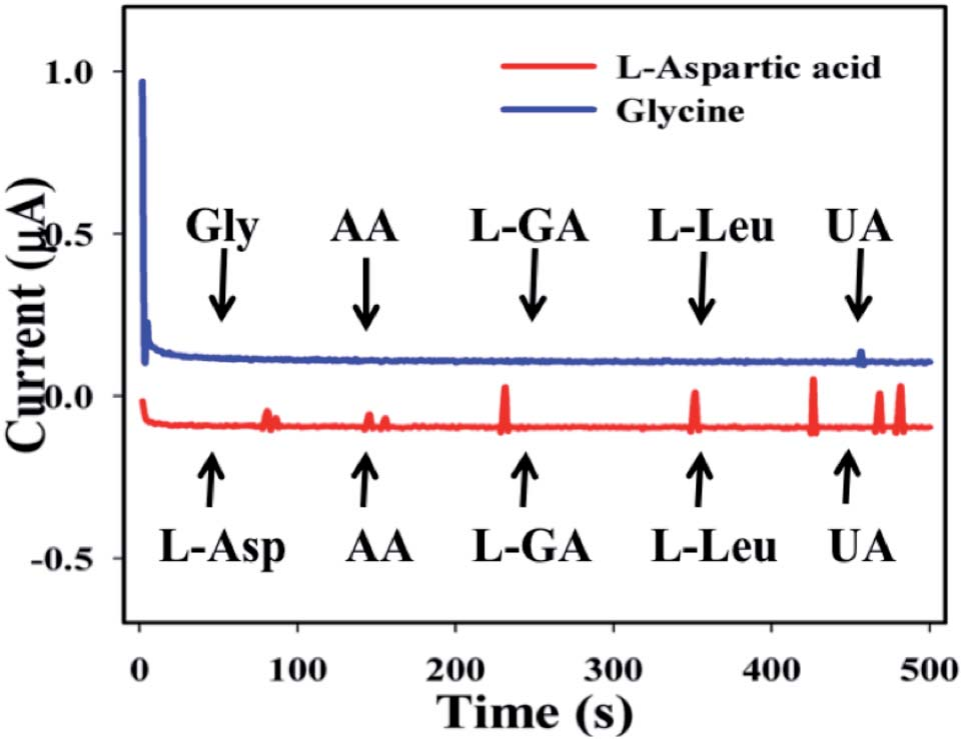
Interference effect of the biomolecules.

The study of interference effects is one of the most significant techniques in analytical science, due to its capability to distinguish interfering agents from biomolecules with similar physiological characteristics.^52–57^ Ascorbic acid (AA), l-glutamic acid (l-GA), l-leucine (l-Leu), and uric acid (UA) were used as interfering constituents in the enzyme free electrochemical l-aspartic acid and glycine detection. Current signals towards the GCE/Fe_3_O_4_@ZnO NPs/Nf sensor upon addition of l-Asp and Gly and interfering biomolecules such as AA, l-GA, l-Leu, and UA (1.0 μM) in PB (10 mL, 100.0 mM, and pH = 6.5) with a specific time range (0–500 s with interval = 100 s) were recorded. In the case of l-aspartic acid, a little interfering effect was observed but for glycine, no effect was found (Fig. 8). It was clear that the proposed sensor (GCE/Fe_3_O_4_@ZnO NPs/Nf) did not demonstrate any notable responses towards interfering agents. Hence, the projected sensor is suitable for detection of l-aspartic acid and glycine with good sensitivity and selectivity.

### 4.4 Real sample analysis

**Fig. 9:**
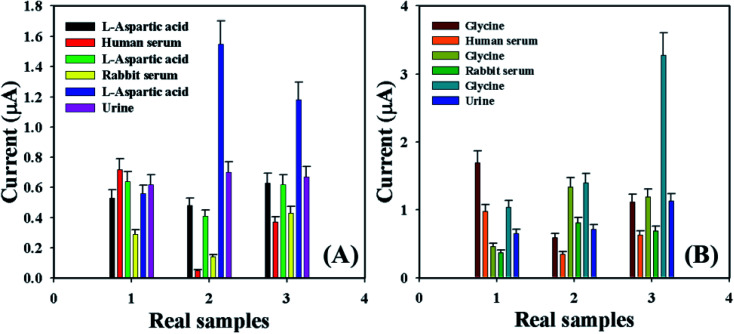
Real sample analysis with error bar 10.0% of (A) l-aspartic acid and (B) glycine.

Accuracy of the current–voltage system was measured by using the GCE/Fe_3_O_4_@ZnO NPs/Nf sensor to identify the l-aspartic acid and glycine concentration in various real samples such as human serum (HS), rabbit serum (RS), and urine (U). A normal adding technique^58–62^ was used in real sample examination in order to enumerate the concentration of l-aspartic acid and glycine. A fixed amount (∼25.0 μL) of the real sample was added and analyzed in PB (10.0 mL, 100.0 mM, and pH = 6.5) using the fabricated GCE/Fe_3_O_4_@ZnO NPs/Nf sensor. The average observed current was measured at calibrated potentials (+0.4 and +0.7 V) of l-aspartic acid and glycine, respectively. The results are shown in Fig. 9 and Table 5 regarding the recognition of l-aspartic acid and glycine in human and rabbit serum and urine, which accurately acknowledged that the current–voltage procedure is dependable, suitable, and ideal for assessment of real samples with the GCE/Fe_3_O_4_@ZnO NPs/Nf customized sensor.

**Table 5:** Analysis of real samples^a^

BM	Real samples	AC (1.0 mM → 1.0 μM → 1.0 nM)	OAC (μA)		FC	*R* (%)	SD (*n* = 3)
			*A*	Real samples			
l-Aspartic acid	HS	1.0	0.53	0.72	1.34	134	0.32
		1.0	0.48	0.05	1.04	104	0.40
		1.0	0.63	0.37	0.59	59	0.60
	RS	1.0	0.64	0.29	0.45	45	0.26
		1.0	0.41	0.14	0.34	34	0.17
		1.0	0.62	0.43	0.69	69	0.03
	U	1.0	0.56	0.62	1.11	111	0.34
		1.0	1.55	0.70	0.45	45	0.05
		1.0	1.18	0.67	0.57	57	0.05
Glycine	HS	1.0	1.70	0.98	0.58	58	0.09
		1.0	0.59	0.35	0.59	59	0.06
		1.0	1.12	0.63	0.56	56	0.07
	RS	1.0	0.46	0.37	0.80	80	0.02
		1.0	1.34	0.81	0.60	60	0.07
		1.0	1.19	0.69	0.58	58	0.05
	U	1.0	1.04	0.65	0.63	63	0.06
		1.0	1.40	0.71	0.51	51	0.05
		1.0	3.28	1.13	0.34	34	0.04

a BM: biomolecules, l-Asp: l-aspartic acid, Gly: glycine, HS: real serum, RS: rabbit serum, U: urine, AC: added concentration, OAC: observed average current, FC: found concentration, *R*: recovery, and SD: standard deviation.

## 5. Conclusions

An easy and dependable wet-chemical technique was used in order to synthesize NPs (ZnO, Fe_3_O_4_, and Fe_3_O_4_@ZnO) in basic medium. The electrochemical properties of the nanoparticles were examined by means of instruments such as UV-vis, FTIR, FESEM, XEDS, XPS and XRD. An uncomplicated fabrication method was applied to modify the GCE with NPs with a coating binder. Selective and sensitive l-aspartic acid and glycine sensors were developed successfully based on a GCE embedded with Fe_3_O_4_@ZnO NPs using the current–voltage system. The sensing performances of the fabricated l-aspartic acid and glycine sensors were found to be good with regards to sensitivity, LOD, LOQ, LDR, response time, reproducible, repeatability, interference effect examination, and real sample analysis. An effective process can be introduced from this new innovation in the perspective of sensor advancement in healthcare fields.

## Author's contribution

MMH and MMR designed the experiments. MMH performed these experiments and wrote the whole manuscript. AMA and MMR supervised this research work and corrected the manuscript. At last, all authors have approved this corrected manuscript to be published in this journal.

## Conflicts of interest

The authors declare that there is no conflict of interest to report in this research work.

## Supplementary Material

RA-010-D0RA03263H-s001
