# Supplementary material for: Simultaneous detection of l-aspartic acid and glycine using wet-chemically prepared Fe3O4@ZnO nanoparticles: real sample analysis
Source: RSC Adv. 2020 May 20;10(33):19276–89. doi: 10.1039/d0ra03263h (PMC9054059; doi:10.1039/d0ra03263h)
Supplement: RA-010-D0RA03263H-s001 [file RA-010-D0RA03263H-s001.pdf]

Electronic Supplementary Material (ESI) for RSC Advances.
This journal is © The Royal Society of Chemistry 2020

Simultaneous detection of L-Aspartic acid and Glycine using wet-chemically prepared Fe_3O_4@ZnO nanoparticles: Real sample analysis†

Mohammad Musarraf Hussain*[abc], Abdullah M. Asiri[ab] and Mohammed M. Rahman*[ab],

[a]*Chemistry Department, Faculty of Science, King Abdulaziz University, Jeddah 21589, P.O. Box 80203, Saudi Arabia*

[b]*Center of Excellence for Advanced Material Research (CEAMR), King Abdulaziz University, Jeddah 21589, P.O. Box 80203, Saudi Arabia*

[c]*Department of Pharmacy, Faculty of Life and Earth Sciences, Jagannath University, Dhaka-1100, Bangladesh*

Electronic supplementary materials (ESM)

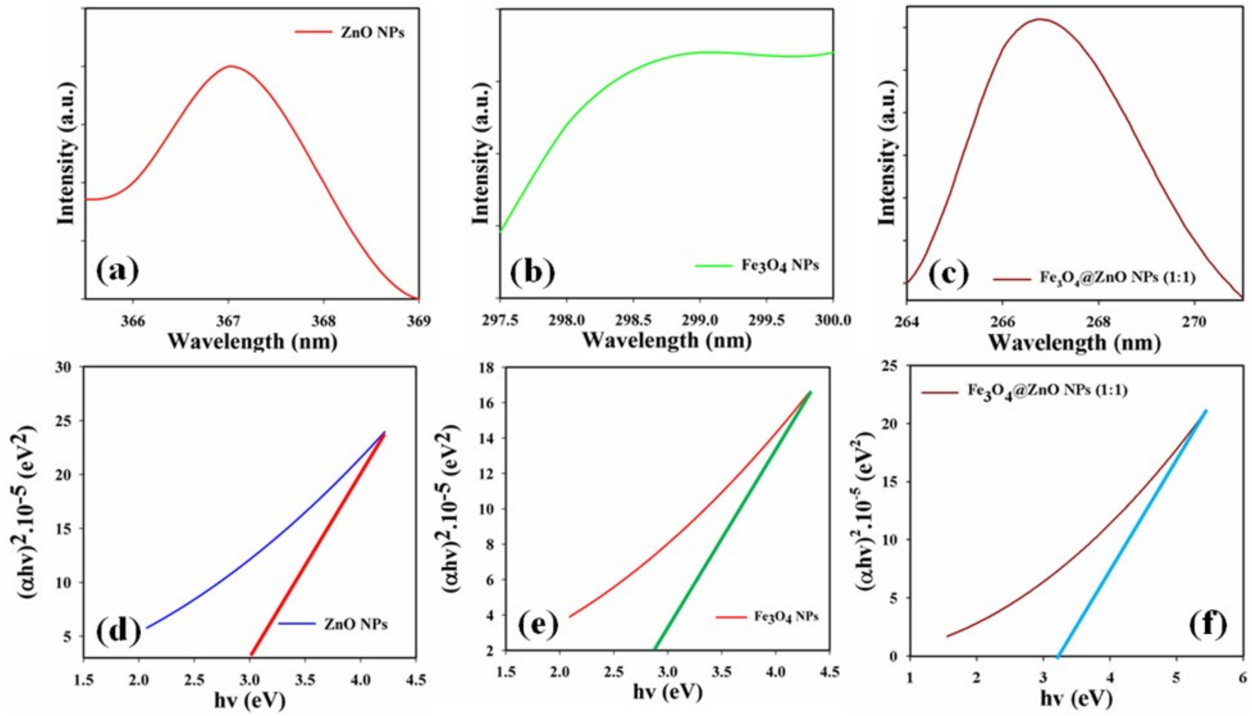

Fig. S1 UV-Visible spectra and band gap energy plot (a-d): ZnO NPs, (b-e) Fe_3O_4 NPs, and (c-f) Fe_3O_4@ZnO NPs (1:1)

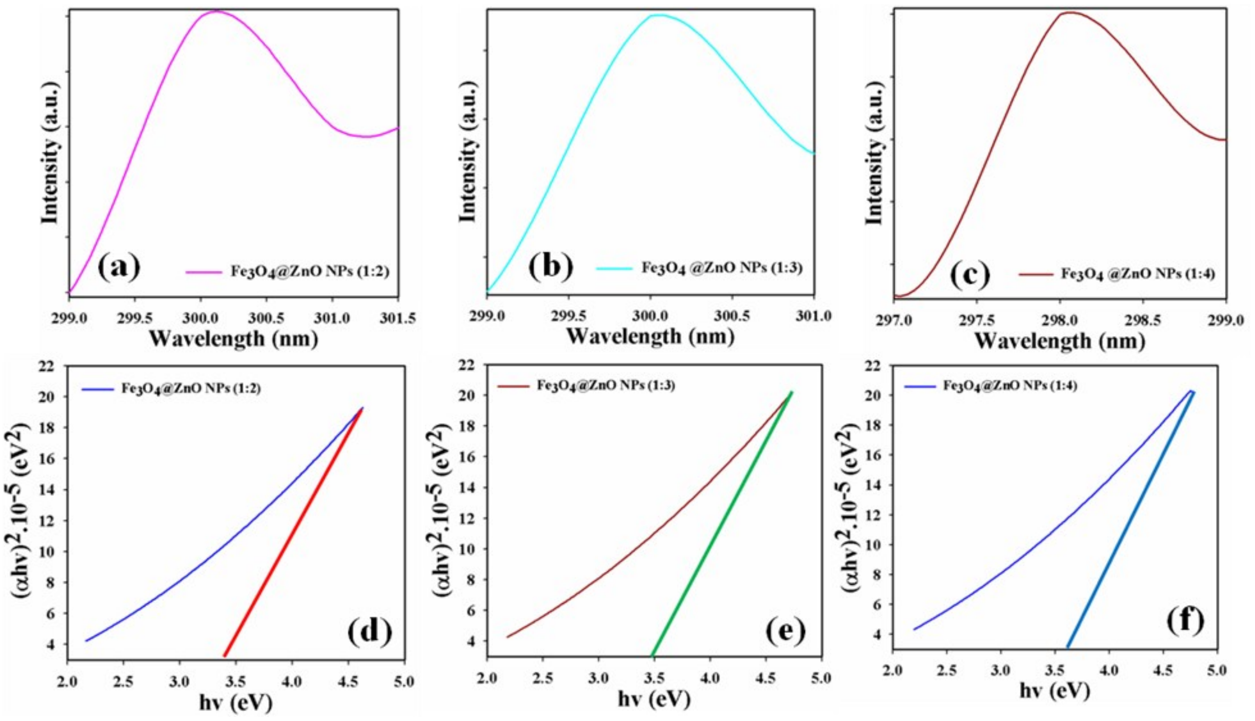

Fig. S2 UV-Visible spectra and band gap energy curves, (a-f) ZnO@Fe_3O_4 NPs (1:2 – 1:4)

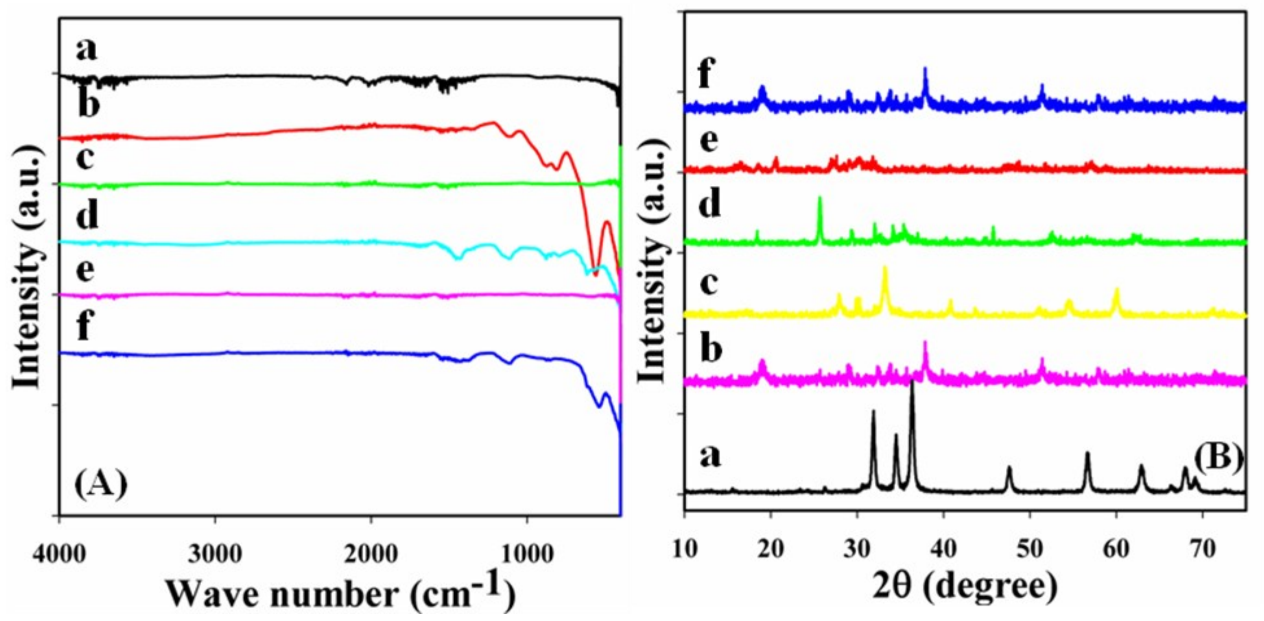

Fig. S3 FTIR and XRD spectra of Fe_3O_4@ZnO NPs, (A-B) [a: ZnO NPs, (b) Fe_3O_4 NPs], A (c-f) [Fe_3O_4@ZnO NPs (1:4 – 1:1)], and B (c-f) [Fe_3O_4@ZnO NPs (1:1 – 1:4)]

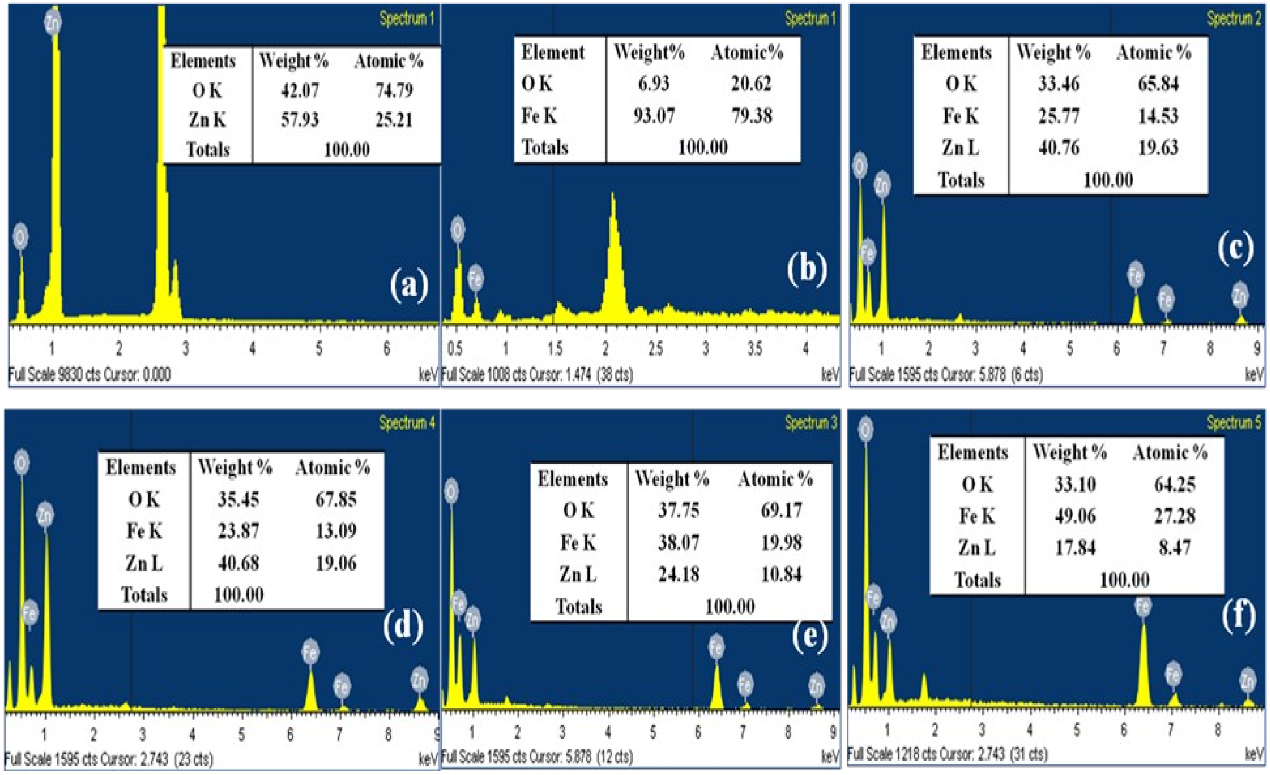

Fig. S4 Elemental analysis, (a) ZnO NPs, (b) Fe_3O_4 NPs, and (c-f) Fe_3O_4@ZnO NPs (1:1 – 1:4)

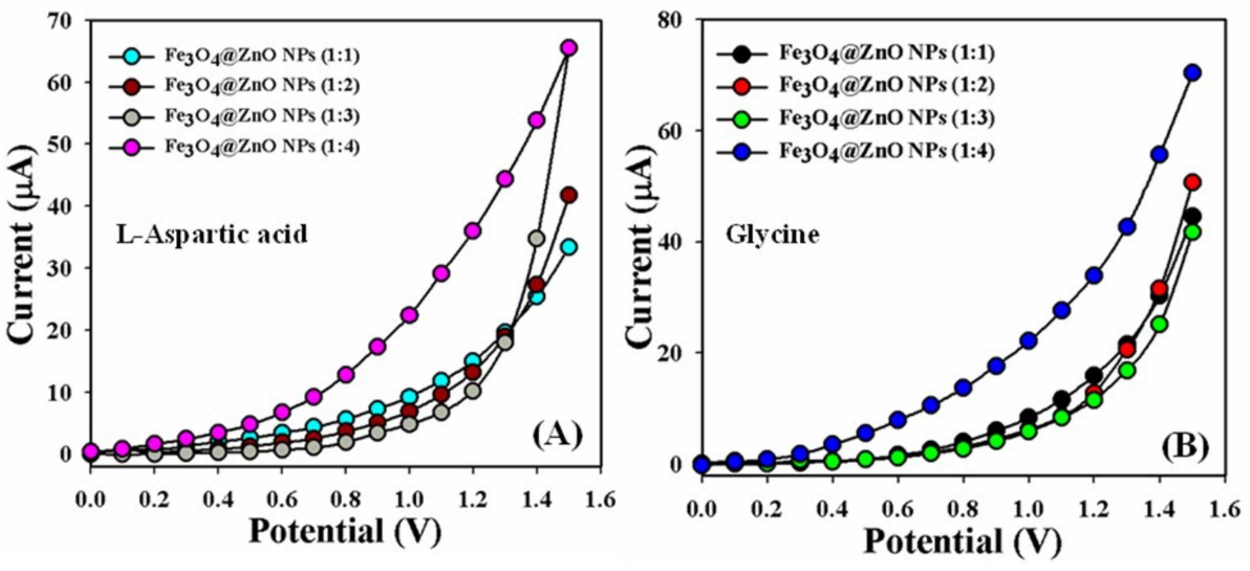

Fig. S5 Selectivity optimization, (A) L-Aspartic acid and (B) Glycine

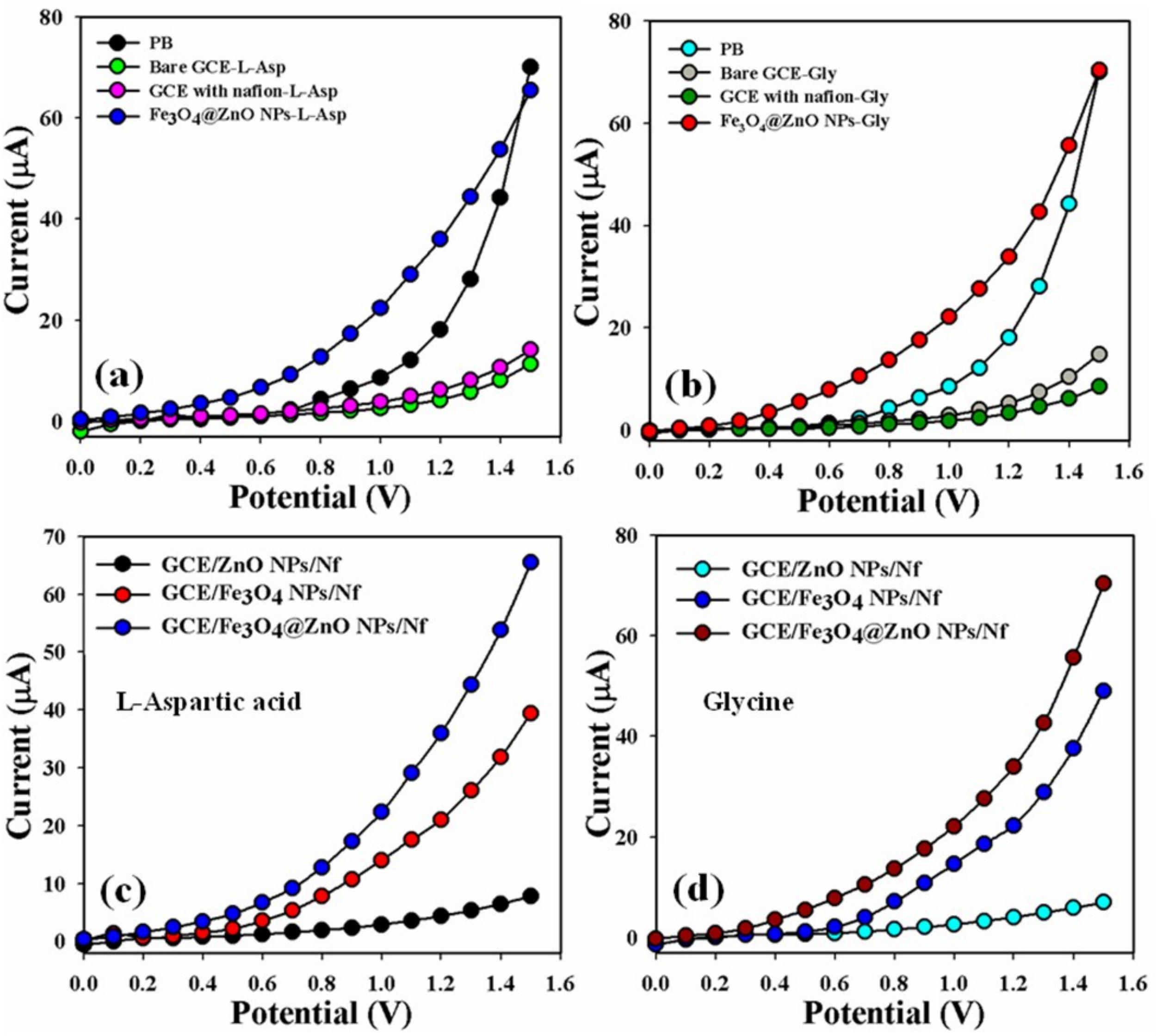

Fig. S6 Absence and presence of bio-molecules and control experiment, (A-C): L-Aspartic acid and (B-D) Glycine

Table S1 Phosphate buffer composition

PB (100 mM, pH)	NaH_2PO_4 (200.0 mM, mL)	Na_2HPO_4 (200.0 mM, mL)	Water (mL)	Amount (mL)
5.7	93.5	6.5	100	200
6.5	68.5	31.5	100	200
7.0	39.0	61.0	100	200
7.5	16.0	84.0	100	200
8.0	5.3	94.7	100	200

Table S2 Reproducibility study of Fe_3O_4@ZnO NPs modified sensor at calibrated potential

Run	L-Aspartic acid			Glycine		
	Current (μA) at + 0.4 V	Reproducibility (%)		Current (μA) at + 0.7 V	Reproducibility (%)	
		Individual	Average		Individual	Average
1	1.43	100		3.73	100	
2	0.53	37		2.45	66	
3	0.52	36	50	1.66	45	58
4	0.58	41		2.01	54	
5	0.63	44		0.62	17	
6	0.55	39		2.45	66	

Here, reproducibility of run 1 has been considered to be 100 %.

Table S3 Repeatability study of Fe_3O_4@ZnO NPs modified sensor at calibrated potential

Run	L-Aspartic acid			Glycine		
	Current (μA) at + 0.4 V	Repeatability (%)		Current (μA) at + 0.7 V	Repeatability (%)	
		Individual	Average		Individual	Average
1	0.70	100		1.20	100	
2	0.70	100		1.13	94	
3	0.70	100	98	1.10	92	90
4	0.67	96		1.01	84	
5	0.70	100		1.01	84	
6	0.65	93		0.99	83	

Here, repeatability of run 1 has been considered to be 100 %.
